# Prognostic factors for perioperative pulmonary events among patients undergoing upper abdominal surgery

**DOI:** 10.1590/S1516-31802007000600003

**Published:** 2007-11-01

**Authors:** Rafael Luis Sakai, Graciela Maria Gera Abrão, José Franscisco Vasques Ayres, Pedro Thadeu Galvão Vianna, Lídia Raquel de Carvalho, Yara Marcondes Machado Castiglia

**Affiliations:** Department of Anesthesiology, Faculdade de Medicina de Botucatu, Universidade Estadual Paulista (Unesp), Botucatu, São Paulo, Brazil

**Keywords:** General anesthesia, Gastrointestinal surgical procedures, Chronic obstructive pulmonary disease, Recovery room, Respiratory insufficiency, Anestesia geral, Procedimentos cirúrgicos do sistema digestório, Doença pulmonar obstrutiva crônica, Sala de recuperação, Insuficiência respiratória

## Abstract

**CONTEXT AND OBJECTIVE:**

The significant relationship between upper abdominal surgery and early (perioperative) pulmonary events was investigated among patients with preoperative pulmonary conditions undergoing general anesthesia.

**DESIGN AND SETTING:**

Retrospective study for which data were obtained prospectively from 1999 to 2004, at a tertiary university hospital.

**METHODS:**

We retrospectively studied 3107 patients over 11 years old presenting American Society of Anesthesiologists (ASA) status I, II or III who underwent upper abdominal surgery under general anesthesia and were discharged to the recovery room. The preoperative conditions analyzed using logistic regression were: age, sex, ASA physical status, congestive heart failure, asthma, chronic obstructive pulmonary disease (COPD), respiratory failure and smoking. The outcomes or dependent variables included intraoperative and postoperative events: bronchospasm, hypoxemia, hypercapnia, prolonged intubation and airway secretion.

**RESULTS:**

Among these patients (1500 males, 1607 females, mean age 48 years, 1088 ASA I, 1402 ASA II and 617 ASA III), there were 80 congestive heart failures, 82 asthmatics, 122 with COPD, 21 respiratory failures and 428 smokers. Logistic regression analysis showed that female sex (p < 0.001), age over 70 years (p < 0.01), smoking (p < 0.001) and COPD (p < 0.02) significantly influenced pulmonary event development, particularly hypoxemia and bronchospasm, at both times but not in the same patients. Asthma and congestive heart failure cases did not present pulmonary events in the recovery room.

**CONCLUSION:**

In upper abdominal surgery under general anesthesia, female sex, age over 70, smoking and COPD were independent risk factors for intra and postoperative pulmonary events.

## RESUMEN

**CONTEXTO E OBJETIVO:**

Associações significativas entre cirurgia do abdome superior e eventos pulmonares do período perioperatório foram investigadas em pacientes com condições pulmonares pré-operatórias submetidos a anestesia geral.0

**TIPO DE ESTUDO E LOCAL:**

Estudo retrospectivo cujos dados foram retirados de banco de dados obtidos prospectivamente de forma protocolada, de 1 de janeiro de 1999 a 31 de dezembro de 2004, em hospital universitário terciário.

**MÉTODOS:**

Estudados 3107 pacientes com mais de 11 anos, American Society of Anesthesiologists (ASA) I, II, III, com cirurgia de abdome superior sob anestesia geral, enviados à sala de recuperação. Condições pré-operatórias analisadas por regressão logística foram: idade, sexo, estado físico ASA, insuficiência cardíaca congestiva, asma, doença pulmonar obstrutiva crônica, insuficiência respiratória e hábito de fumar. Os resultados estudados, ou variáveis dependentes, incluíram eventos intra- e pós-operatórios: broncoespasmo, hipoxemia, hipercapnia, intubação prolongada e secreção de vias aéreas.

**RESULTADOS:**

Dos 3.107 pacientes: 1.540 eram homens, 1.649 mulheres, tinham média de 48 anos, 1088 ASA I, 1402 ASA II, 617 ASA III, com insuficiência cardíaca havia 80, asma, 82, doença pulmonar obstrutiva, 122, insuficiência respiratória, 21, hábito de fumar, 428. Pela regressão logística, sexo feminino (p < 0.001), idade maior que 70 anos (p < 0.01), hábito de fumar (p < 0.001) e doença pulmonar obstrutiva crônica (p < 0.02) influenciaram significativamente o desenvolvimento de eventos pulmonares, principalmente hipoxemia e broncospasmo, em ambos os períodos, mas não nos mesmos pacientes. Asma e insuficiência cardíaca não se associaram com eventos pulmonares na sala de recuperação.

**CONCLUSÃO:**

Em cirurgia do abdome superior sob anestesia geral, sexo feminino, idade maior que 70 anos, hábito de fumar e doença pulmonar obstrutiva crônica foram fatores de risco independentes para a ocorrência de eventos pulmonares intra- e pós-operatórios.

## INTRODUCTION

Systematic and structured efforts are needed in order to improve the quality of anesthetic care, based on examination of the circumstances surrounding undesired events. Pulmonary complications during the pre, intra and postoperative periods have been a matter of concern for many years with regard to anesthesia.

Because of the introduction of more insoluble inhalation anesthetics, which enable tracheal tube removal in the operating room and the use of pulse oximeters in clinical practice, the factors that may influence the occurrence of postoperative pulmonary complications, such as age, gender, weight, intraoperative opioid administration, smoking habits, duration of anesthesia and preexisting heart and lung disease have been studied extensively with a new viewpoint. Mitchell et al.^1^ described a sample of 148 patients in which 11% (16 patients) developed postoperative pulmonary complications. Upper abdominal surgery under general or spinal anesthesia had been performed on 11 (69%) of these 16 patients and on 20 (15%) of the 132 who remained without pulmonary complications, but this difference lost significance in multivariate analysis.

A prospective trial^2^ was conducted on 408 patients who were analyzed preoperatively and underwent surgery under general anesthesia, with the incision above the navel level. These patients were then followed up postoperatively with regard to pulmonary complications. Chronic pulmonary disease, comorbidity and surgery lasting more than 210 minutes were found to be the three major clinical risk factors for pulmonary complications. In another study on patients undergoing general anesthesia, the severity of arterial desaturation and the incidence of hypoxemia in the early postoperative period were closely related to the surgical site location, such that these were greatest in cases of thoracoabdominal surgery, less for upper abdominal surgery and least for peripheral surgery.^3^

Arozullah et al. developed and validated a preoperative risk index for predicting postoperative respiratory failure. More than 180,000 patients were enrolled in two phases of the study and it was found that the highest risk of developing postoperative respiratory failure was related to the type of surgery. Upper abdominal surgery was the third worst type, only preceded by abdominal aortic aneurysm and thoracic surgery. The greatest patient-specific risk factors related to general health status, renal and fluid status and respiratory status.^4^ Another study found that age greater than 56 years, surgery lasting more than 210 minutes and pneumopathy were important markers for postoperative pulmonary complications following elective upper abdominal surgery under general anesthesia.^5^ It can therefore be seen that more studies on the overall situation are needed.

Patients undergoing general anesthesia for thoracoabdominal surgery may go into the intensive care unit immediately after the procedure, but those undergoing general anesthesia for upper abdominal surgery are generally extubated before going to the recovery room. Induction of anesthesia causes disruption to the normal activity of the respiratory muscles that may continue into the postoperative period, even if early extubation is possible. One matter that could be questioned here is the correlation between pulmonary events and preoperative pulmonary conditions.

## OBJECTIVE

The aim of this study was to evaluate whether some preoperative pulmonary conditions among patients undergoing general anesthesia for upper abdominal (gastrointestinal) surgery can be correlated with pulmonary events in the intraoperative period and postoperative period in the recovery room.

## METHODS

### Study definitions

The following definitions were used in this study:

#### Congestive heart failure:

previously diagnosed by clinicians, including abnormal tolerance to exercise due to dyspnea or fatigue, orthopnea, paroxysmal nocturnal dyspnea, increased jugular venous pressure, pulmonary rales on physical examination, cardiomegaly and pulmonary vascular engorgement;

#### Asthma:

clinical condition reported by the patient, with or without the use of drugs during the attacks;

#### Chronic obstructive pulmonary disease (COPD):

previously diagnosed by clinicians, or diagnosed by the anesthesiologist during the pre-anesthesia assessment, among patients with specific clinical features, such as reports of increasing shortness of breath over several years with chronic cough, poor exercise tolerance and evidence of airway obstruction, overinflated lungs and impaired gas exchange, which might or might not require bronchodilator therapy or present functional disability;

#### Respiratory failure:

presence of signs and symptoms of the underlying disease combined with those of hypoxemia, such as oxygen saturation < 90%, dyspnea, cyanosis, restlessness, confusion, anxiety, delirium, tachypnea, tachycardia, hypertension, cardiac arrhythmia and tremor; and those of hypercapnia such as peripheral and conjunctival hyperemia, hypertension, tachycardia, and tachypnea;

#### Smoking habit:

patients who had smoked more than 100 cigarettes during their entire lives and those who were current smokers at the time of the pre-anesthesia evaluation for index surgery were defined as presenting a smoking habit;

#### Bronchospasm:

wheezing on auscultation;

#### Hypoxemia:

hemoglobin saturation < 90%;

#### Hypercapnia:

end tidal carbon dioxide pressure > 45 mmHg or > than the patients’ normal value, for those with COPD;

#### Pulmonary edema:

diminished hemoglobin saturation and evidence of fluid accumulation in the lungs from clinical examination, which was treated with intravenously administered diuretic therapy);

#### Augmented airway secretion:

chest ronchi, increased quantity of sputum in the tracheal tube or development of a productive cough.

The events studied in the recovery room were: bronchospasm, hypoxemia (defined as hemoglobin saturation < 90%, in 21% oxygen, 60 minutes after extubation; and with the need for oxygen supplementation, via mask), hypercapnia, prolonged intubation (when extubation occurred in the recovery room at least 30 minutes after the patient arrived), and augmented airway secretion.

### Study population

The protocol for this study was reviewed and approved by the ethics committee of our medical school. We retrospectively studied 3107 consecutive patients who were over 11 years old. Patients were only included in this study if they were classified as presenting American Society of Anesthesiologists (ASA) physical status I, II or III, and underwent upper abdominal (gastrointestinal) surgery under general anesthesia administered by inhalation (isoflurane or sevoflurane with an opioid — fentanyl, alfentanil or sufentanil — and with or without nitrous oxide) or intravenously (propofol and an opioid with or without nitrous oxide). These patients were discharged to the recovery room of our teaching hospital between January 1, 1999, and December 31, 2004. There were no sample losses.

Medical data were collected from the database of the Department of Anesthesiology of our medical school. This database provided prospective preoperative, intraoperative and postoperative (recovery room) information on patients anesthetized by our staff, and consists of a quality assurance database from contemporaneous medical records, customized data sheets and laboratory result records. Residents were responsible for these anesthetic procedures, while always under full direct supervision by the professors of anesthesiology.

It has been mandatory for our staff to conduct pre-anesthesia assessments since 1970. This is the time to decide whether the patient is fit for anesthesia and to adapt the anesthetic procedure to the patient's condition. In the present study, five clinical conditions evaluated during the pre-anesthesia assessment were recorded: congestive heart failure, asthma, COPD, respiratory failure and smoking habits (no data could be obtained for the amount of tobacco consumed). Associations between these preoperative conditions (with and without) and the pulmonary events found intraoperatively and postoperatively in the recovery room (yes and no) were evaluated. The possible intraoperative pulmonary events were defined as aspiration of gastric content, bronchospasm (defined as wheezing on auscultation), hypoxemia (intraoperative hemoglobin saturation < 90% in 100% O_2_), hypercapnia (end tidal carbon dioxide pressure > 45 mmHg or > than the patients’ normal value, for those with COPD), pulmonary edema (diminished hemoglobin saturation and evidence of fluid accumulation in the lungs) and augmented airway secretion. The pulmonary events in the recovery room were: bronchospasm, hypoxemia (defined as hemoglobin saturation < 90%, in 21% oxygen, 60 minutes after extubation, and with the need for oxygen supplementation, via mask), hypercapnia, prolonged intubation and augmented airway secretion.

### Statistical analysis

In order to investigate the influence of previous clinical conditions (classified as with and without) on intraoperative and postoperative (recovery room) pulmonary events (classified as yes and no), the data were put into 2 × 2 tables with the observed values and the percentages of yes occurrences for the patients with and without previous clinical conditions (values in parentheses). Chi-squared analysis, with calculation of χ^2^ and p, or the Fisher exact test, with direct calculation of p, was used as appropriate, in order to ascertain any differences between the percentages of intraoperative and postoperative pulmonary events among the patients with and without the previous conditions.^5^ When p ≤ 0.05, percentages were considered to be significantly different.

Preoperative conditions such as age, sex, ASA physical status, congestive heart failure, asthma, COPD, respiratory failure and smoking habits were entered into logistic regression analysis with the intraoperative and postoperative events as the dependent variables.

## RESULTS

Out of the 3107 patients who met our inclusion criteria, 671 were urgent cases and 397 were emergency cases; 1500 (48.28%) were male and 1607 (51.72%) were female, with a mean age of 48 years and mode of 47 years. ASA physical status I was presented by 1088 patients (35.0%), II by 1402 (45.1%) and III by 617 (19.9%). The mean body mass index was 25.13. The mean duration of the surgery was 166 ± 91 minutes, and the mean duration of anesthesia was 214 ± 97 minutes. Among the patients studied, 80 (2.57%) presented congestive heart failure diagnosed preoperatively; 82 (2.64%), asthma; 122 (3.93%), COPD; 21 (0.68%), respiratory failure; and 428 (13.78) had smoking habits (Table 1). The mean ages, in years, of the patients with these preoperative conditions were: congestive heart failure, 69 ± 12; asthma, 48 ± 16; COPD, 62 ± 13; respiratory failure, 53 ± 19; and smoking habits, 52 ± 15.

**Table 1:** Characteristics of patients who underwent upper abdominal surgery

Category		Total (%)
**Age (years)**	
ߓ 11–20	204 (6.6)
ߓ 21–30	388 (12.5)
ߓ 31–40	456 (14.7)
ߓ 41–50	562 (18.1)
ߓ 51–60	613 (19.7)
ߓ 61–70	512 (16.5)
ߓ 71–80	309 (9.9)
ߓ > 81	63 (2.0)
**ASA status**	
ߓ I	1088 (35.0)
ߓ II	1402 (45.1)
ߓ III	617 (19.9)
**Congestive heart failure**	80 (2.57)
**Asthma**	82 (2.64)
**COPD**	122 (3.93)
**Respiratory failure**	21 (0.68)
**Smoking habit**	428 (13.78)
**Mean duration of surgery (min.)**	166 ± 91
**Mean duration of anesthesia (min.)**	214 ± 97

ASA = American Society of Anesthesiologists; COPD = chronic obstructive pulmonary disease.

In these patients, the intraoperative pulmonary events were aspiration of gastric content (11 cases), bronchospasm (58 cases), hypoxemia (48 cases) and hypercapnia (15 cases). Three patients who presented intraoperative bronchospasm had COPD and smoking habits. The same was noticed in relation to hypoxemia. There were statistically significant associations (p < 0.05) between congestive heart failure and hypoxemia; asthma and aspiration of gastric content, bronchospasm and hypercapnia; COPD and bronchospasm and hypoxemia; and smoking habits and bronchospasm and hypoxemia. Respiratory failure did not present any associations with intraoperative events (Table 2).

**Table 2:** Association between preoperative clinical conditions and intraoperative pulmonary event occurrence

Preoperative clinical condition	Pulmonary events	Aspiration (%) (11 cases)	Bronchospasm (%) (58 cases)	Hypoxemia (%) (48 cases)	Hypercapnia (%) (15 cases)
**Congestive heart failure**	With	0 (0.00)	1 (1.25)	5 (6.25)*	0 (0.00)
n = 80 (2.6%) 69 ± 12 years	Without	11 (0.36)	57 (1.88)	43 (1.42)	15 (0.50)
**Asthma**	With	3 (3.66)*	9 (10.98)*	1 (1.22)	2 (2.44)*
n = 82 (2.6%) 48 ± 16 years	Without	8 (0.26)	49 (1.62)	47 (1.55)	13 (0.43)
**COPD**	With	1 (0.82)	6 (4.92)*	5 (4.10)*	2 (1.64)
n = 122 (3.9%) 62 ± 13 years	Without	10 (0.34)	52 (1.74)	43 (1.44)	13 (0.44)
**Respiratory failure**	With	1 (4.76)	1 (4.76)	0 (0.00)	0 (0.00)
n = 21 (0.7%) 53 ± 19 years	Without	10 (0.32)	57 (1.85)	48 (1.56)	15 (0.49)
**Smoking habit**	With	1 (0.23)	18 (4.21)*	12 (2.80)*	2 (0.47)
n = 428 (13.8 %) 52 ± 15 years	Without	10 (0.37)	40 (1.49)	36 (1.34)	13 (0.49)

* p < 0.05; COPD = chronic obstructive pulmonary disease.

The postoperative pulmonary events in the recovery room were bronchospasm (9 cases), hypoxemia (143 cases), hypercapnia (8 cases), prolonged intubation (10 cases) and augmented airway secretion (9 cases). The patients who presented bronchospasm in the recovery room were not among those who presented bronchospasm during the intraoperative period. Only three patients diagnosed with hypoxemia during the intraoperative period had hypoxemia in the recovery room. One of these had a smoking habit. The other two did not present any preoperative pulmonary conditions examined in this study. Asthma and congestive heart failure did not present any significant associations with these occurrences in the recovery room. COPD presented statistically significant associations with bronchospasm and augmented airway secretion; respiratory failure, with prolonged intubation; and smoking habits, with hypoxemia (Table 3).

**Table 3:** Association between preoperative clinical conditions and pulmonary event occurrence in the recovery room

Preoperative clinical condition	Pulmonary events	Bronchospasm (%) (9 cases)	Hypoxemia (%) (143 cases)	Hypercapnia (%) (8 cases)	Prolonged intubation (%) (10 cases)	Augmented airway secretion (%) (9 cases)
**Congestive heart failure**	With	0 (0.00)	3 (3.75)	1 (1.25)	0 (0.00)	0 (0.00)
n = 80 (2.6%) 69 ± 12 years	Without	9 (0.30)	140 (4.63)	7 (0.23)	10 (0.33)	9 (0.30)
**Asthma**	With	1 (1.22)	4 (4.88)	0 (0.00)	0 (0.00)	0 (0.00)
n = 82 (2.6%) 48 ± 16 years	Without	8 (0.26)	139 (4.60)	8 (0.26)	10 (0.32)	9 (0.30)
**COPD**	With	4 (3.28)*	6 (4.92)	0 (0.00)	1 (0.82)	3 (2.46)*
n =122 (3.9%) 62 ± 13 years	Without	5 (0.17)	137 (4.59)	8 (0.27)	9 (0.30)	6 (0.20)
**Respiratory failure**	With	0 (0,00)	0 (0.00)	0 (0.00)	1 (4.76)*	0 (0.00)
n = 21 (0.7%) 53 ± 19 years	Without	9 (0.29)	143 (4.63)	8 (0.26)	9 (0.29)	9 (0.29)
**Smoking habit**	With	1 (0.23)	30 (7.01)*	0 (0.00)	2 (0.47)	1 (0.23)
n = 428 (13.8%) 52 ± 15 years	Without	8 (0.30)	113 (4.22)	8 (0.30)	8 (0.30)	8 (0.30)

* p < 0.05; COPD = chronic obstructive pulmonary disease.

By means of logistic regression analysis, female sex (OR 1.55, 95% CI 1.20-2.00, p = 0.001), old age (OR 1.65, 95% CI 1.12-2.43, p = 0.011), smoking habit (OR 1.72, 95% CI 1.26-2.36, p = 0.001) and COPD (OR 1.87, 95% CI 1.11-3.15, p = 0.019) were found to significantly influence the development of pulmonary events during the intraoperative and postoperative periods (Table 4).

**Table 4:** Preoperative clinical conditions associated with the development of pulmonary events during the intraoperative and postoperative periods, according to logistic regression

Variable	Coefficient	Standard error	Z	p	Odds ratio (OR)	95% CI (confidence interval)
Constant	-3.35144	0.248305	-13.50	0.000		
Sex (female)	0.437314	0.129951	3.37	0.001	1.55	[1.20; 2.00]
Age (in decades)						
[40-60]	0.299963	0.160255	1.87	0.061	1.35	[0.99; 1.85]
[60-70]	0.297074	0.196757	1.51	0.131	1.35	[0.92; 1.98]
≥ 70	0.501835	0.197508	2.54	0.011	1.65	[1.12; 2.43]
Smoking habit	0.544807	0.160062	3.40	0.001	1.72	[1.26; 2.36]
COPD	0.626441	0.266214	2.35	0.019	1.87	[1.11; 3.15]

COPD = chronic obstructive pulmonary disease.

## DISCUSSION

There is substantial postoperative mortality among upper abdominal surgery patients, and an association has been established between perioperative coma and death and certain anesthesia management factors like the characteristics of the intraoperative and postoperative anesthetic care that is provided.^6^ Some preoperative conditions may be highly associated with intraoperative and postoperative pulmonary events among patients undergoing upper abdominal surgery. Therefore, it would be preferable to predict which patients these would be, so as to improve their outcomes.

The present study found through multivariable analysis that certain patient-specific risk factors, such as female sex, age over 70 years, smoking habit and COPD, were important in relation to the development of pulmonary events during the perioperative period of upper abdominal surgical procedures. When populations of older patients undergo surgery, the in-hospital postoperative complications must be minimized, particularly those involving the pulmonary and renal systems.^7^

During the intraoperative period, changes occur in breathing patterns, and these may persist into the postoperative period.^8^ The disruption of the normal activity of the respiratory muscles begins with the induction of anesthesia and may continue into the postoperative period. In some cases, the lack of coordination is more important than the overall lack of activity. Surgical trauma may be an additional effect of these intraoperative changes, along with a residual anesthetic effect. Although this factor has not been studied enough, it has evident importance in relation to the new anesthetics, with their rapid discharge from the body.

The effect of surgical trauma is more pronounced following thoracic and abdominal surgery. This arises from at least three mechanisms: functional disruption of the respiratory muscles by incisions, even after surgical repair; postoperative pain that causes voluntary limitation of respiratory motion; and stimulation of the viscera that decreases phrenic motor neuron output and changes other respiratory muscle activity. Multiple afferent pathways mediate this reflex.^9^ Surgical trauma may also disrupt the normal coordination of respiratory muscle action, such that the functional residual capacity and vital capacity will persistently decrease, with lung atelectasis that leads to pneumonia, although this progression has not been conclusively shown.^8^

These postoperative changes can be partially ameliorated by using endoscopic techniques to minimize surgical trauma.^10^ However, the laparoscopic cholecystectomy procedure, for instance, still stimulates the abdominal viscera through gallbladder traction, and the pulmonary mechanics will be affected. In the recovery room, the first appearance of atelectasis would be hypoxemia. A systematic review of the literature on interventions to prevent postoperative pulmonary complications following non-cardiothoracic surgery affirmed that it was unclear whether laparoscopic procedures reduced the risk of clinically significant pulmonary complications, even though their use was supported by spirometric, postoperative pain and length-of-stay data.^11^

For our patients, asthma represented an important risk factor only for developing events intraoperatively. Good preoperative performance and inhalation anesthesia using bronchodilator agents, such those currently in use, may be the factors that improved the outcome in the recovery room.

During the intraoperative period, pulmonary aspiration of gastric content was a significant occurrence for our asthmatic patients. This is a pulmonary event within anesthetic practice that may result in acute lung injury: it is life-threatening and has consequences that will be seen in the intensive care unit.^12^ However, asthma is not among the diseases or conditions that might affect gastric emptying or fluid volume, such as pregnancy, obesity, diabetes, hiatal hernia, gastroesophageal reflux disease, ileus or bowel obstruction, emergency care or enteral tube feeding. Therefore, it might be that these patients had some of the comorbidities or conditions described earlier.

In another study, the existing medical records were reviewed in order to determine the frequencies and risk factors for perioperative pulmonary complications among a cohort of patients with asthma. In undergoing anesthesia and surgery, these patients showed surprisingly low frequencies of perioperative bronchospasm and laryngospasm. These complications did not lead to severe respiratory outcomes in most patients, but the frequency of complications was greater among older patients and among those with active asthma.^13,14^ The asthmatic patients described in the present study were not elderly.

In the present study, the patients with a smoking habit had a statistically significant association with intraoperative bronchospasm and hypoxemia and also with hypoxemia in the recovery room. To examine the effect of preoperative smoking behavior on perioperative pulmonary complications, a prospective study was conducted among patients who were scheduled for non-cardiac elective surgery. This concluded that current smoking was associated with a nearly sixfold increase in the risk of such complications. Reduction in smoking less than one month before surgery was not associated with decreased risk of perioperative pulmonary complications.^15^

With regard to COPD, the intraoperative pulmonary complications observed in the present study were intraoperative bronchospasm and hypoxemia and in the recovery room, bronchospasm. Another study examined the incidence of different perioperative pulmonary complications and their associated risk factors, among patients with severe COPD. The specific risk factors were determined for each complication by analyzing the potential preoperative and intraoperative risk factors.^16^ It was found that the pulmonary factors alone did not predict the likelihood of perioperative pulmonary complications among patients with severe COPD patients. Multiple logistic regression showed that composite scoring systems such as ASA physical status were the best preoperative predictors of perioperative pulmonary complications, probably because they included both pulmonary and non-pulmonary factors. The authors of that study postulated that, during the intraoperative period, avoidance of general anesthesia with tracheal intubation might decrease the risk of postoperative bronchospasm. However, this is not applicable to patients undergoing upper abdominal surgery.

Congestive heart failure presented a significant positive association only with intraoperative hypoxemia. Millions of intra-abdominal operations are performed each year, and these procedures are associated with relatively high rates of pulmonary and cardiac complications.^17^ One study suggested that pulmonary events may be more common than cardiac complications and be associated with longer hospitalization, occurring in combination with cardiac complications in a substantial proportion of the cases.^17^ In preoperative evaluations, cardiac risk assessment may be based on extensive research, but this is not so for pulmonary operative risk. Cardiac operative risk has been more rigorously and extensively studied than has pulmonary operative risk. Poor standardization and definition of perioperative pulmonary complications are believed to be among the problems.^18^

To identify predictors for postoperative pulmonary complications, a study was conducted on a population of 278 patients who were mainly hypertensive and diabetic, and 60% of these patients underwent abdominal surgery. Only 16 patients had pulmonary complications and those with underlying asthma or chronic bronchitis were found to be at increased risk. Pulmonary function tests also did not help to identify the patients who were at higher risk. Many of the complications occurred in patients who also had other types of postoperative morbidity, thus suggesting that predicting and preventing postoperative cardiac morbidity may be the best approach towards reducing postoperative pulmonary morbidity.^19^

In order to analyze the performance of variables commonly used in predicting perioperative pulmonary complications among patients undergoing non-thoracic surgery, a systematic review of the literature in English from 1966 to 2001 was conducted.^20^ Seven studies fulfilled the inclusion criteria. The definition of perioperative pulmonary complications differed among these studies, and the incidence ranged from 2% to 19%. Of the 28 preoperative or operative predictors that were evaluated in these seven studies, 16 were associated significantly with perioperative pulmonary complications, although only two (duration of anesthesia and postoperative nasogastric tube placement) were significant in more than one study. The positive (2.2 to 5.1) and negative (0.2 to 0.8) likelihood ratios for these 16 variables suggested that they only had modest predictive value. Hypercarbia and reduced spirometry values were not independently associated with increased risk of perioperative pulmonary complications. The authors concluded that few studies had rigorously evaluated the performance of pre­operative or operative variables for predicting perioperative pulmonary complications. They suggested that more prospective studies with independent and blind comparisons of these variables with postoperative outcomes needed to be performed.^20^

Smetana et al.^21^ systematically reviewed the literature on preoperative pulmonary risk stratification prior to non-cardiothoracic surgery to investigate its value in relation to postoperative pulmonary complications. There was good evidence supporting the use of certain patient-related risk factors, including advanced age, ASA class II or higher, functional dependence, COPD and congestive heart failure. Nevertheless, among the procedure-related risk factors, abdominal surgery appeared in fourth place.

The present study showed that there was higher incidence of pulmonary events during the intraoperative period than during the postoperative period (recovery room). All of the preoperative conditions, except for respiratory failure, were associated with hypoxemia first, and bronchospasm secondly. Perhaps respiratory failure has its own solution through tracheal intubation. Surprisingly, asthma and congestive heart failure did not present any pulmonary event in the recovery room. The new approaches towards treating these conditions, and the fact that only in real life-threatening situations is surgery considered for such patients (especially with regard to congestive heart failure), may be two of the explanations for these results. The other conditions presented the expected events: COPD, bronchospasm, augmented airway se­cre­ti­on, respiratory failure, prolonged in­tu­ba­tion, smoking habits and hypoxemia.

## CONCLUSION

In cases of upper abdominal surgery under general anesthesia, female sex, age older than 70, smoking habits, and COPD were independent risk factors for the occurrence of intra- and postoperative pulmonary events.
